# Comparison of Antioxidant Capacity and Muscle Amino Acid and Fatty Acid Composition of Nervous and Calm Hu Sheep

**DOI:** 10.3390/antiox12020459

**Published:** 2023-02-11

**Authors:** Jinying Zhang, Yifan Zhang, Jiasheng Wang, Hengyu Jin, Shuhan Qian, Peigen Chen, Mengzhi Wang, Ning Chen, Luoyang Ding

**Affiliations:** 1 College of Animal Science and Technology, Yangzhou University, Yangzhou 225009, China; 2 State Key Laboratory for Sheep Genetic Improvement and Healthy Production, Xinjiang Academy of Agricultural and Reclamation Science, Shihezi 832061, China

**Keywords:** Hu sheep, temperament, oxidation resistance, amino acid, fatty acid

## Abstract

This study determined the effect of temperament on antioxidant capacity and the relationship between antioxidant capacity and the contents of amino acids (AA) and fatty acids (FA) in muscle of Hu sheep. Organ and muscle samples of five calm and five nervous Hu sheep were collected to determine the antioxidant capacity and the contents of AA and FA in muscle tissue. The concentrations of malondialdehyde (MDA) and superoxide excretion enzyme (SOD) in muscle and intestinal tissue of calm Hu sheep were lower than those of nervous Hu sheep (*p* < 0.01), and the activity of glutathione peroxidase (GSH-Px) in liver of calm Hu sheep was significantly higher than that of nervous Hu sheep (*p* = 0.050). The content of AA of calm Hu sheep was higher than that of nervous Hu sheep, especially the content of reductive amino acids, which was significantly higher than that of nervous Hu sheep (*p* = 0.029). Fatty acid content of nervous Hu sheep was higher than that of calm type, and saturated fatty acid content was significantly higher than that of calm type (*p* = 0.001). The SOD content in muscle tissue was positively correlated with the contents of aspartic acid (Asp), alanine (Ala) and lysine (Lys). Catalase (CAT) activity was positively correlated with Ala content. There was a significant positive correlation between total antioxidants (T-AOC) and glutamate (Glu) (*p* < 0.05). MDA concentration was positively correlated with lauric acid (C12:0), triseconic acid (C13:0), myristic acid (C14:0) content (*p* < 0.01), and ginkgo acid (C15:0) content. The total antioxidants (T-AOC) was negatively correlated with stearic acid (C18:0) (*p* < 0.05). Our conclusion is that the antioxidant capacity of calm Hu sheep is superior to that of nervous Hu sheep, which may be due to the higher AA (especially reductive amino acids (Arg, Lys, Ala and Glu)) content in the muscle and the lower FA (especially SFA) content, which improve the antioxidant capacity of the organism and allow for further exploration of the mechanisms by which animal temperament affects antioxidant performance.

## 1. Introduction 

In order to fulfill the rapidly expanding human demand for animal products, intensive farming can boost productivity, but the resulting rise in a number of stimuli elements has a detrimental effect on animal health and production performance [1]. Animal welfare research is currently experiencing a slow increase, and there is a relationship between animal temperament and animal welfare. Animals can perceive the stressors present in a new habitat by exhibiting a wide variety of behavioral reactions, which are thought to reflect the animal’s temperament [2,3]. Antioxidant capacity is a crucial metric for measuring the degree of oxidative stress. Some preliminary research has discovered that nervous animals are more sensitive to pressure, which can easily lead to an increase in lipid peroxide, resulting in oxidative damage to the body [4]. However, the effect of temperamental differences on the antioxidant capacity of tissues and organs and the specific mechanism of action need to be further explored. Various flavor precursors (amino acids (AA) and fatty acids (FA)) in mutton have been shown to have effects on meat quality and to be related to antioxidant capacity [5]. However, no correlation analysis has been reported among animal temperament, antioxidant capacity, and AA and FA. This severely limited further investigation into the relationship or mechanism of action of AA and FA on antioxidant capability. Therefore, we hypothesized that temperament could modify the oxidation resistance through AA and FA composition in muscle. For this purpose, the current work was conducted to provide some basic data for animal health and quality breeding by analyzing the effects of temperament differences on antioxidant performance, muscle AA and FA content, and their regulatory mechanisms, and at the same time to provide new thinking directions for breeding high-quality animal breed technology and regulation technology.

## 2. Materials and Methods

All animal experimental projects were accredited and authorized by the Experimental Animal Care and Use Committee of Yangzhou University, China (SXXY 2015-0054). 

### 2.1. Test Animals and Feeding Management

All animal procedures were in accordance with and approved by the Animal Ethical Committee of Yangzhou University. According to the nutrient requirements of fattening sheep with a body weight of 40 kg and a daily gain of 400 g in NRC (2007), total mixed diet (TMR) was formulated to feed experimental Hu sheep. The basic diet composition and nutrient levels are shown in Table S1. Forty Hu sheep 5.5 months old weighing (40.0 ± 3.6) Kg were selected, and then according to Bickell’s open-field [6], temperament was assessed by testing the behavioral responses of individual sheep to the presence of humans estimated. Briefly, the candidate sheep entered a test arena (3.3 m × 7 m), which was divided into 4 sectors of identical size through a door that was situated at the opposite end of the arena to a pen that contained a group of ewes that was visible from the arena. The locomotor activity of the candidate sheep was recorded as crosses by counting the number of sectors the sheep crossed, and the number of high-pitch bleats was recorded as bleats during a three-minute test. The higher scores for crosses and bleats have been found to be associated with a higher response to the presence of humans and is thus considered as a more nervous temperament.

### 2.2. Sample Collection

At the end of the test, the test Hu sheep were slaughtered, and six parts of tissues were separated, including muscle, liver, spleen, pancreas, kidney and intestine, which were respectively placed in 1.5 mL freezing tubes, quickly frozen in liquid nitrogen, and stored at −80 °C.

### 2.3. Determination of Antioxidant Indexes in Each Tissue

The antioxidant indexes (total antioxidants (T-AOC), catalase (CAT), glutathione peroxidase (GSH-Px), superoxide excretion enzyme (SOD) and malondialdehyde (MDA)) were determined. SOD activity was determined by xanthine oxidase method, MDA content was determined by thiobarbituric acid method, T-AOC was determined by the total antioxidant capacity detection kit (ABTS method), GSH-Px activity and CAT activity were determined by the colorimetric method. All the kits used for index determination were purchased from Nanjing Jiancheng Bioengineering Institute and tested according to the kit instructions.

### 2.4. Determination of Amino Acids and Fatty Acids in Muscle of Hu Sheep

For the determination of amino acid content in the muscle of Hu sheep, refer to GB 5009.124–2016 “Determination of Amino Acid in Food”. Appropriate samples were taken and 10–15 mL 6 mol/L hydrochloric acid solution was added, and 3–4 drops of phenol were frozen, hydrolyzed, filtered and dried. The sample determination solution was prepared by adding 1.0–2.0 mL pH 2.2 sodium citrate to dissolve the solution. The amino acid concentration in the sample determination solution was calculated by using the external standard method of amino acid automatic analyzer [7]. According to GB 5009.168–2016 “Determination of Fatty Acids in Food”, the sample was weighed at 0.1–10 g, the sample was hydrolyzed by the acid hydrolysis method, after hydrolysis, 10 mL of 95% ethanol was added to extract the hydrolysate to obtain the fat extract, then the fat was saponified and methyl esterified, and then methyl ester was prepared. The single fatty acid methyl ester standard solution and the mixed standard solution of fatty acid methyl ester were injected into the gas chromatograph, and the chromatographic peaks were qualitatively determined. Finally, the fatty acid standard solution and the sample determination solution were injected into the gas chromatograph under the above chromatographic conditions, and the chromatographic peak area was quantified [8].

### 2.5. Statistical Analysis

The normality of variables was analyzed using the Shapiro–Wilk test of SPSS 22. T-test was used to analyze the influence of temperament on measurement parameters. *p ≤* 0.05 indicates significant difference.

## 3. Results

### 3.1. Effects of Temperament on the Antioxidant Capacity of Muscles and Organs of Hu Sheep

#### 3.1.1. Effects of Temperament on the Antioxidant Capacity of Hu Sheep Muscles

The MDA concentration in the muscles of nervous Hu sheep was significantly higher than that of calm Hu sheep (*p* < 0.05). The activities of SOD and T-AOC in calm Hu sheep were higher than those in nervous Hu sheep (Table 1).

#### 3.1.2. Effects of Temperament on the Antioxidant Capacity of Liver of Hu Sheep

The activity of GSH-Px in the liver of calm Hu sheep was significantly higher than that of nervous Hu sheep (*p* = 0.050) (Table 2).

#### 3.1.3. Effects of Temperament on the Antioxidant Capacity of Spleen of Hu Sheep

The activity of SOD and GSH-Px in the spleen of calm Hu sheep tended to be higher than that of nervous Hu sheep (Table 3).

#### 3.1.4. Effects of Temperament on the Antioxidant Capacity of Kidney in Hu Sheep

The activity of SOD in the kidneys of nervous Hu sheep tended to be higher than that of calm Hu sheep. The calm Hu sheep tended to have higher T-AOC activity (Table 4).

#### 3.1.5. Effects of Temperament on the Antioxidant Capacity of Pancreatic in Hu Sheep

The activity of SOD in the pancreas of calm Hu sheep was higher than that of nervous Hu sheep. The T-AOC activity of calm Hu sheep tended to be higher (Table 5).

#### 3.1.6. Effects of Temperament on the Intestinal Antioxidant Capacity of Hu Sheep

The intestines collected in this study included the duodenum, jejunum, ileum, and cecum. The concentration of MDA in the intestinal tract of nervous Hu sheep was significantly higher than that of calm Hu sheep (*p* = 0.005). The activity of SOD in the intestinal tract of nervous Hu sheep was significantly higher than that of calm Hu sheep (*p* < 0.01) (Table 6).

### 3.2. Comparison of the Antioxidant Capacity of Different Organs of Hu Sheep

**Figure 1:**
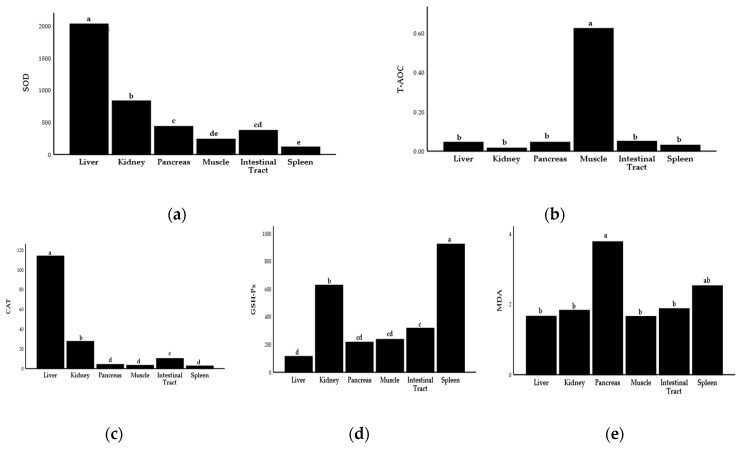
Comparison of antioxidant capacity in different organs and tissues of Hu sheep. (**a**) Comparison of SOD activity in different organs. (**b**) Comparison of T-AOC in different organ tissues. (**c**) Comparison of CAT activity in different organ tissues. (**d**) Comparison of GSH-Px activity in different organs and tissues. (**e**) Comparison of MDA content in different organs and tissues. The values are expressed as the mean with standard error represented by vertical bars (*n* = 10). SOD, superoxide dismutase; MDA, malondialdehyde; GSH-Px, glutathione peroxidase; CAT, catalase; T-AOC, total antioxidant capacity. ^a,b,c,d,e^ Mean values in the columns without a common letter differ (*p ≤* 0.05).

The activities of SOD and CAT in liver tissue of Hu sheep were significantly higher than those in other organs, but the contents of T-AOC in muscle tissue were significantly higher than those in other organs, and the contents of MDA in pancreas and spleen were significantly higher than those in other tissues (*p* < 0.05). In general, the antioxidant capacity of all organs of Hu sheep was liver, muscle > kidney > intestine tract, spleen, and pancreas (Figure 1; Supplementary Table S2).

### 3.3. Effects of Temperament on Amino Acid and Fatty Acid Contents in Muscle Tissue of Hu Sheep

**Figure 2:**
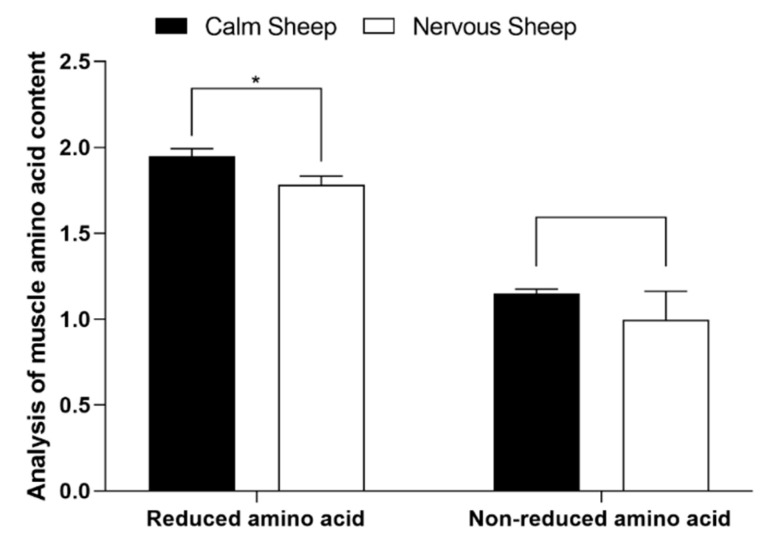
Effects of different temperaments on the amino acid contents of two kinds of muscles in Hu sheep (%). The values are expressed as the mean with standard error represented by vertical bars (*n* = 5). * indicate that there were significant (*p ≤* 0.05) differences between the two temperaments.

The contents of glutamate (Glu) and isoleucine (Ile) in the muscles of calm Hu sheep were significantly higher than those of nervous Hu sheep (*p* < 0.001), while the content of serine (Ser) was significantly higher than that of nervous Hu sheep (*p* = 0.016) (Table 7). AA were classified into reductive amino acids (arginine (Arg), lysine (Lys), alanine (Ala), Glu)) and non-reductive amino acids (serine (Ser), glycine (Gly), Ile, aspartic (Asp), threonine (Thr)). The results showed that the content of reductive amino acids in the muscles of calm Hu sheep was significantly higher than that of nervous Hu sheep (*p* = 0.029) (Figure 2; Supplementary Table S3).

**Figure 3:**
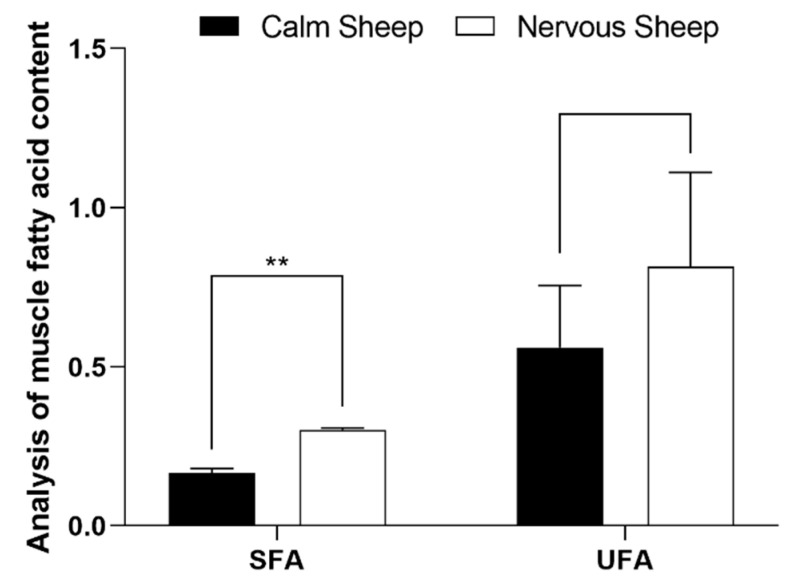
Effects of different temperaments on the contents of two kinds of muscle fatty acids in Hu sheep (%). The values are expressed as the mean with standard error represented by vertical bars (*n* = 5). ** indicate that there were significant (*p ≤* 0.05) differences between the two temperaments.

The contents of lauric acid (C12:0) and thirteen carbonic acid (C13:0) in muscle tissue of nervous Hu sheep were significantly higher than those of calm Hu sheep (*p* < 0.05), and the contents of myristic acid (C14:0) and palmitic acid (C16:0) were significantly higher than those of calm Hu sheep (Table 8). The content of monounsaturated fatty acids (MUFA) and polyunsaturated fatty acids (PUFA) in muscle tissue of the nervous type was higher than that of the calm type. The content of saturated fatty acids (SFA) in muscle of the nervous type was significantly higher than that of the calm type (*p* = 0.001) (Figure 3; Supplementary Table S4).

### 3.4. Correlation Analysis of Amino Acid and Fatty Acid Contents and Antioxidant Capacity in the Muscle of Hu Sheep

**Figure 4:**
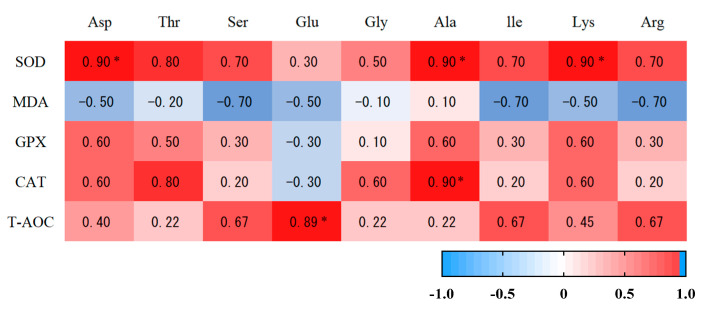
The correlation analysis of amino acids and antioxidant capacity in the muscle of Hu sheep. * indicates that there were significant (*p ≤* 0.05) differences between the two temperaments.

**Figure 5:**
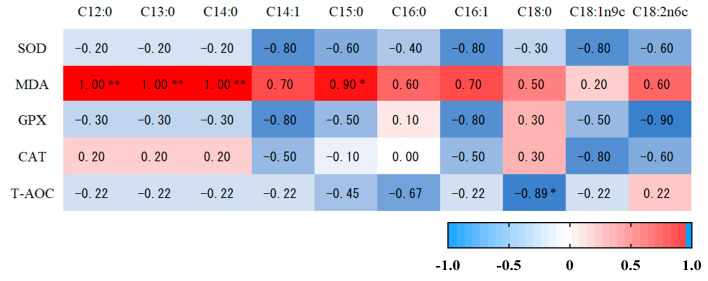
The correlation analysis of muscular fatty acids and antioxidant capacity of Hu sheep. * and ** indicates that there were significant (*p ≤* 0.05) differences between the two temperaments.

The SOD activity in muscle tissue was positively correlated with ASP content, Ala content, and Lys content (*p* < 0.05). There was a significant positive correlation between CAT activity and Ala content (*p* < 0.05). There was a significant positive correlation between T-AOC content and Glu content (*p* < 0.05) (Figure 4). MDA concentration in muscle tissue was positively correlated with the contents of C12:0, C13:0 and C14:0 (*p* < 0.01) and was positively correlated with fifteen carbonic acid (C15:0). T-AOC was negatively correlated with stearic acid (C18:0) (*p* < 0.05) (Figure 5).

## 4. Discussion

### 4.1. Comparison of Antioxidant Capacity between Calm and Nervous Hu Sheep

Animals may experience oxidative stress in intense production due to a variety of environmental and social variables. For instance, a higher breeding density increases the likelihood of free radical production in the body, which leads to oxidative damage. In addition, bacterial infections, transit stress, cold and heat stress, animal fights, and inadequate hygiene in the feeding area can all result in stress responses and oxidative damage [9].

Generally speaking, lower MDA concentrations and stronger antioxidant performance are associated with higher levels of T-AOC, SOD, GSH-Px, and CAT activity in the body [10]. Studies have shown that people with schizophrenia, who frequently resemble animals with neurotic temperaments, have rather significant levels of oxidative damage [11]. Stressed animals have considerably higher MDA contents, plasma levels of 5-hydroxytryptamine (5-HT), and oxidative stress levels [12], which is related to the loss of intestinal mucosal barrier function caused by intestinal stress [13]. 5-HT is a key neurotransmitter involved in mood regulation [14] and is intimately linked to the body’s antioxidant capability. When the body is stressed, it can stimulate the intestinal sympathetic nervous system and release norepinephrine (NE), which acts on the main cells synthesizing 5-HT through intestinal chromaffin cells and 5-HT-capable neurons, thus increasing the release of 5-HT [15]. It was found in experiments that the content of 5-HT in calm pigs was lower, while the content of SOD and GSH-Px in the intestines was higher [16].

We examined the changes in antioxidant capacity in the various organs of Hu sheep. One of the essential metrics for measuring the body’s antioxidant effect is the T-AOC of organs and tissues. SOD, a key antioxidant enzyme, is crucial for maintaining the proper ratio of antioxidants to oxidants in the body. The body can be shielded from free radical poisoning by this enzyme’s ability to scavenge anti-superoxide anion free radicals (O^2-^) [17]. The liver is the primary organ of detoxification in vertebrates and contains a variety of enzymes. Animal livers are susceptible to a variety of poisons because after a toxin enters the body and is delivered to the liver, it undergoes redox interactions with liver enzymes to lower its toxicity. Most vertebrates’ skeletal muscle contains the water-soluble endogenous dipeptide carnosine, which is produced in vivo by the enzyme carnosine synthase from β-alanine and L-histidine. Studies have demonstrated that carnosine has a natural potential for antioxidants and can considerably reduce the oxidation of lipids brought on by free radicals and metal ions. When preparing meat, adding the right amount of carnosine can prevent fat oxidation and preserve meat color. Reactive oxygen (ROS) is produced in large quantities in mitochondria, which can result in mutations in the mitochondrial DNA [18]. By safeguarding mitochondrial activity, decreasing ROS production, and preventing 4-hydroxynonenal from binding to elastin, carnosine can postpone the onset of aging and lessen the harm resulting from lipid peroxidation [19]. Carnosine also decreases the production of important intermediate reactive carbonyl compounds or prevents them from attaching to proteins, which minimizes the production of melanin and lipofuscin and limits the formation of further oxidative products. Carnosine can considerably diminish the rise in protein carbonyl groups brought on by aging, as was established by the administration of carnosine to D-galactose-induced aging mice. This might be connected to carnosine’s anti-inflammatory and anti-glycosylation properties [20].

### 4.2. Comparison of Amino Acids and Fatty Acids in Muscle of Hu Sheep with Different Temperaments

Amino acid content and composition in muscle are important indexes to evaluate its nutritional value and an important factor affecting meat quality. Meat flavor is mainly formed by Ile, Gly, Ser, Glu, Ala and Arg [21], among which Glu is the main substance constituting meat umami and produces flavor through the Maillard reaction and reducing sugar [22], thus improving the palatability of mutton. Arg can enhance mitochondrial biosynthesis, activate adenosine 5’-monophosphonic acid (AMP)-activated protein kinase (AMPK) signal, increase nitric oxide (NO) synthesis, promote the transformation of fast muscle fibers to slow muscle fibers, increase intramuscular fat deposition [23], and improve meat tenderness and meat juiciness [24]. Greater economic value in the actual production can come from more delicate and better-colored flesh. FA in muscle have a significant impact on meat’s softness and juiciness as well as its nutritional value [25]. FA consist of PUFA, SFA, and MUFA [26]. Mutton with a lower proportion of SFA in total fatty acids [27] had higher quality and a lower prevalence of cardiovascular disease in animals [28]. Meat is susceptible to oxidative rancidity during storage and processing when the amount of unsaturated fatty acids (UFA) in the muscle is high, which affects the taste and flavor of meat [27]. The flavor of the mutton can be enhanced during production by altering the stearic acid and other mutton odor markers in SFA [29]; however, the high palmitic acid content of SFA will raise the prevalence of cardiovascular disorders [30]. In conclusion, nervous Hu sheep have inferior softness, flavor, and nutritional value to calm Hu sheep, which may be directly related to the amount of AA and FA in muscle.

An animal’s temperament also affects its behavior. Grumpy animals will have a greater response to stress, prone to display violent abnormal characteristics. For example, nervous animals will respond more to artificially placed shock rods and have a marked tendency to behave aggressively [31]. The study compared the behavioral differences between the two temperaments, and the results showed that the variation frequency of the nervous type was 2.4 times that of the calm type, indicating that the nervous type may be more prone to psychological depression and behavioral shift [32]. Meanwhile, the feeding time of calm Hu sheep is longer than that of nervous Hu sheep, which is more conducive to the fattening production of Hu sheep [32]. Animal behavior is somewhat influenced by the AA and FA contents. For instance, in the heat, a lack of arginine causes pecking, walking, and hostility in chickens. In the brain, Arg can be converted into Glu, and both compounds have the ability to stimulate the release of growth hormone and control animal feeding behavior [33]. According to research on animal pica behavior, fluctuations in the blood’s Ile levels may be responsible for the behavior’s occurrence in Angus beef cattle [34]. Additionally, if Arg is not fully involved in gluconeogenesis, it will not be able to supply the body with adequate energy to meet its needs, which would likely result in pica in cattle [35].

### 4.3. Relationship between Antioxidant Capacity and Amino Acid Content of Hu Sheep with Different Temperaments

Amino acid composition and content were closely related to antioxidant capacity. In the study on the effect of antioxidant capacity on Yili horses, it was found that the addition of AA in the diet could promote the endocrine SOD of Yili horses and reduce the free radical oxidation reaction [36]. Improving the efficiency of protein degradation into AA in muscle can alleviate the problem of reduced meat tenderness caused by slaughter stress, thus improving pork flavor [37]. Currently known amino acid antioxidant mechanisms include: direct scavenging of free radicals and reactive oxygen species, inhibition of metal ions to produce oxygen free radicals, synergistic action with other antioxidants, and reaction with oxidizing lipids to play an antioxidant role [38].

In addition to improving the antioxidant capacity by reducing the production of peroxides [39], Arg can also be metabolized to produce NO, improve the activity of antioxidant enzymes in the body, and reduce the content of superoxides [40], thus improving the immune and antioxidant performance of the body [9]. Thr is involved in lymphocyte proliferation, monocyte, cytokine, and immunoglobulin synthesis [41] and reduces free radical content by stabilizing and chelating metal ions [42]. Other studies have found that Lys also plays a beneficial role in body growth and development, immune function, free radical scavenging, and other aspects [43]. Both Glu and Gly are precursors of GSH synthesis, which directly affects the antioxidant capacity. Similarly, Ile can improve the activity of GSH in serum and reduce the concentration of MDA, thus improving antioxidant performance [44].

In order to obtain a more general rule, Arg, Lys, Ala and Glu were classified as reductive amino acids. Lys and Arg are both antioxidant amino acids. According to research, Arg has some reducing power because free radicals can interact with the electrons that the guanidine group of arg provides, interrupting the chain reaction of free radicals [45]. Ala is one of the hydrophobic amino acids and has a potent antioxidant effect [46]. This can be the case because the side chain group of Ala is an alkyl group, which can function as an electron donor to stop the reactivity of free radicals, and it has a certain capacity to provide electrons and a high reducing power [47,48]. As one of the polar amino acids, Glu is negatively charged and can scour free radicals by improving the water solubility of the lipophilic part of free radical substances [49].

In conclusion, the calm Hu sheep may improve the antioxidant performance of the body by increasing the amino acid content in muscle, especially the reductive amino acids content. Compared with the nervous Hu sheep, the health status, immune ability and meat quality of the calm Hu sheep are more prominent. However, the mechanism of how temperament regulates the antioxidant performance of Hu sheep through AA needs further study.

### 4.4. Relationship between Antioxidant Capacity and Fatty Acid Content of Hu Sheep with Different Temperaments

**Figure 6:**
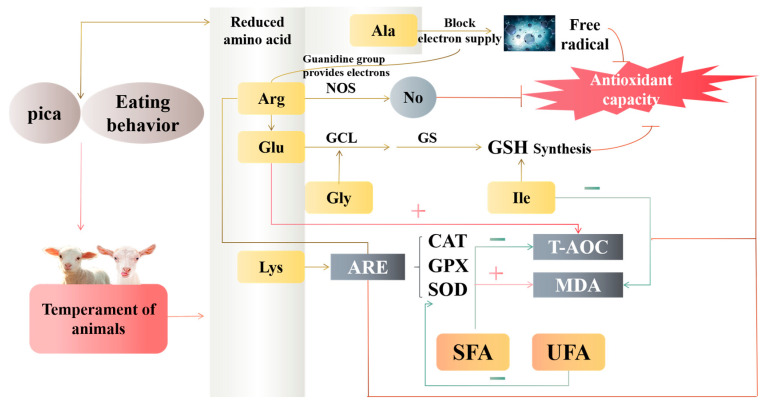
Regulation of antioxidant capacity and mechanism of amino acids and fatty acids in muscle by temperament in animals. Glu, glutamate; Gly, glycine; Ala, alanine; Ile, isoleucine; Lys, lysine; Arg, arginine; ARE, antioxidant reaction element; SFA, saturated fatty acids; UFA, unsaturated fatty acid; SOD, superoxide dismutase; MDA, malondialdehyde; GSH-Px, glutathione peroxidase; CAT, catalase; T-AOC, total antioxidant capacity; GCL, glutamylcysteine ligase; GS, glutamine synthase; NOS, nitric oxide synthase.

Antioxidant activity interacts with saturated fatty acid level. When the body has an excessive amount of SFA, the concentration of MDA rises, which causes apoptosis [50] and exacerbates oxidative stress [51]. Altering the mitochondrial oxidative respiratory chain’s regular operation might also make oxidative stress worse [52]. On the other hand, the body’s reaction to stress has an impact on how much FA is present. The amount of SFA in the milk fat of heat-stressed cows and the serum of stressed rats both increased [53,54]. Because the muscle of calm Hu sheep contains less FA and has a less robust stress response, it may have a better antioxidant capacity than the muscle of nervous Hu sheep. Figure 6 displays the study’s key findings.

## 5. Conclusions

In summary, the antioxidant capacity of the calm Hu sheep was higher than that of the nervous Hu sheep, with better health and production performance and more outstanding meat quality. Antioxidant capacity was correlated with the levels of AA and FA. Specifically, we report for the first time that antioxidant capacity can be improved in calm Hu sheep through higher levels of reductive amino acids (Arg, Lys, Ala and Glu) and lower levels of SFA, thereby improving animal health and production performance and that AA and FA are also associated with behavioral expression of animal temperament. However, the specific mechanisms by which temperament regulates antioxidant capacity through muscle amino acid and fatty acid content need further investigation.

## Figures and Tables

**Table 1:** Effects of different temperaments on the antioxidant indexes of Hu sheep muscle ^1^.

Item	Calm Sheep	Nervous Sheep	SEM	*p* Value
SOD, U/mL	263.63	211.07	69.76	0.506
MDA, mmol/mL	1.19	2.11 *	0.20	0.019
GSH-Px, U/mL	231.78	240.26	110.20	0.943
CAT, U/mL	2.83	3.61	1.06	0.518
T-AOC, U/mL	0.60	0.65	0.05	0.444

SOD, superoxide dismutase; MDA, malondialdehyde; GSH-Px, glutathione peroxidase; CAT, catalase; T-AOC, total antioxidant capacity. ^1^ Mean values with their standard errors of the mean (SEM), *n* = 5 in each group. * indicates that there were significant (*p ≤* 0.05) differences between the two temperaments.

**Table 2:** Effects of different temperaments on the antioxidant indexes of Hu sheep liver ^1^.

Item	Calm Sheep	Nervous Sheep	SEM	*p* Value
SOD, U/mL	2 049.50	2 018.53	241.88	0.906
MDA, mmol/mL	1.55	1.76	0.66	0.771
GSH-Px, U/mL	160.34 *	66.41	29.44	0.050
CAT, U/mL	113.25	114.45	10.83	0.919
T-AOC, U/mL	0.03	0.06	0.01	0.205

SOD, superoxide dismutase; MDA, malondialdehyde; GSH-Px, glutathione peroxidase; CAT, catalase; T-AOC, total antioxidant capacity. ^1^ Mean values with their standard errors of the mean (SEM), *n* = 5 in each group. * indicates that there were significant (*p ≤* 0.05) differences between the two temperaments.

**Table 3:** Effects of different temperaments on the antioxidant indexes of Hu sheep spleen ^1^.

Item	Calm Sheep	Nervous Sheep	SEM	*p* Value
SOD, U/mL	165.85	65.05	92.81	0.473
MDA, mmol/mL	3.19	1.85	1.73	0.580
GSH-Px, U/mL	1 007.75	837.88	204.75	0.453
CAT, U/mL	2.20	2.93	1.04	0.598
T-AOC, U/mL	0.03	0.03	0.20	1.000

SOD, superoxide dismutase; MDA, malondialdehyde; GSH-Px, glutathione peroxidase; CAT, catalase; T-AOC, total antioxidant capacity. ^1^ Mean values with their standard errors of the mean (SEM), *n* = 5 in each group.

**Table 4:** Effects of different temperaments on the antioxidant indexes of Hu sheep kidney ^1^.

Item	Calm Sheep	Nervous Sheep	SEM	*p* Value
SOD, U/mL	751.65	917.45	80.53	0.132
MDA, mmol/mL	1.30	2.35	0.90	0.325
GSH-Px, U/mL	723.60	529.55	242.11	0.563
CAT, U/mL	28.76	26.17	8.54	0.811
T-AOC, U/mL	0.02	0.01	0.00	0.219

SOD, superoxide dismutase; MDA, malondialdehyde; GSH-Px, glutathione peroxidase; CAT, catalase; T-AOC, total antioxidant capacity. ^1^ Mean values with their standard errors of the mean (SEM), *n* = 5 in each group.

**Table 5:** Effects of different temperaments on the antioxidant indexes of Hu sheep pancreas ^1^.

Item	Calm Sheep	Nervous Sheep	SEM	*p* Value
SOD, U/mL	468.54	404.71	58.89	0.470
MDA, mmol/mL	4.56	2.98	0.52	0.202
GSH-Px, U/mL	214.12	217.43	33.07	0.927
CAT, U/mL	3.74	4.46	0.82	0.445
T-AOC, U/mL	0.05	0.04	0.01	0.205

SOD, superoxide dismutase; MDA, malondialdehyde; GSH-Px, glutathione peroxidase; CAT, catalase; T-AOC, total antioxidant capacity. ^1^ Mean values with their standard errors of the mean. (SEM), *n* = 5 in each group.

**Table 6:** Effects of different temperaments on the antioxidant indexes of Hu sheep intestinal ^1^.

Item	Calm Sheep	Nervous Sheep	SEM	*p* Value
SOD, U/mL	231.14	518.17 **	11.35	<0.01
MDA, mmol/mL	1.01	2.73 **	0.23	0.005
GSH-Px, U/mL	207.49	425.71	75.25	0.062
CAT, U/mL	7.25	12.94	4.80	0.321
T-AOC, U/mL	0.06	0.04	0.02	0.196

SOD, superoxide dismutase; MDA, malondialdehyde; GSH-Px, glutathione peroxidase; CAT, catalase; T-AOC, total antioxidant capacity. ^1^ Mean values with their standard errors of the mean (SEM), *n* = 5 in each group. ** indicate that there were significant (*p ≤* 0.05) differences between the two temperaments.

**Table 7:** Analysis of muscle amino acid content in Hu sheep with different temperaments (%) ^1^.

Item	Calm Sheep	Nervous Sheep	SEM	*p* Value
Glu	3.24 **	2.79	0.02	<0.01
Ser	0.82 *	0.75	0.02	0.016
Gly	0.94	0.53	0.43	0.517
Ala	1.23	1.22	0.02	0.799
Ile	0.98 **	0.86	0.01	0.003
Asp	1.99	1.86	0.09	0.252
Thr	1.01	0.99	0.03	0.576
Lys	1.96	1.87	0.10	0.443
Arg	1.38	1.25	0.04	0.062

Glu, glutamate; Ser, serine; Gly, glycine; Ala, alanine; Ile, isoleucine; Asp, aspartic; Thr, threonine; Lys, lysine; Arg, arginine. ^1^ Mean values with their standard errors of the mean (SEM), *n* = 5 in each group. * and ** indicate that there were significant (*p ≤* 0.05) differences between the two temperaments.

**Table 8:** Analysis of muscle fatty acid content in Hu sheep with different temperaments (%) ^1^.

Item	Calm Sheep	Nervous Sheep	SEM	*p* Value
SFA				
C12:0	0.0032	0.0063 *	0.0010	0.047
C13:0	0.0015	0.0031 *	0.0005	0.051
C14:0	0.07	0.24 **	0.01	0.001
C15:0	0.0174	0.0360	0.0075	0.088
C16:0	0.39	0.85 **	0.05	0.002
C18:0	0.52	0.68	0.06	0.075
MUFA				
C14:1	0.0050	0.0076	0.0020	0.294
C16:1	0.08	0.13	0.04	0.290
C18:1n9c	1.84	2.62	0.79	0.397
PUFA				
C18:2n6c	0.26	0.28	0.03	0.597

SFA, saturated fatty acids; C12:0, lauric acid; C13:0, thirteen carbonic acid; C14:0, myristic acid; C15:0, fifteen carbonic acid; C16:0, palmitic acid; C18:0, stearic acid; MUFA, monounsaturated fatty acids; C14:1, cardamomic acid; C16:1, palmitoleic acid; C18:1n9c, oleic acid; PUFA, polyunsaturated fatty acids; C18:2n6c, linoleic acid. ^1^ Mean values with their standard errors of the mean (SEM), *n* = 5 in each group. * and ** indicate that there were significant (*p ≤* 0.05) differences between the two temperaments.

## Data Availability

All data relevant to the study are included in the article or are uploaded as supplementary information. Data are available on reasonable request. Data generated and analyzed during this study are available from the corresponding author on reasonable request.

## Supplementary Materials

The following supporting information can be downloaded at: https://www.mdpi.com/article/10.3390/antiox12020459/s1, Table S1: Composition and nutrient levels of the basal diet (DM basis); Table S2: Comparison of antioxidant capacity in different organs and tissues of calm Hu sheep; Table S3: Effects of different temperament on amino acid contents of two kinds of muscles in Hu sheep (%); Table S4: Effects of different temperament on contents of two kinds of muscle fatty acids in Hu sheep (%).
