# Supplementary material for: Comparison of Antioxidant Capacity and Muscle Amino Acid and Fatty Acid Composition of Nervous and Calm Hu Sheep
Source: Antioxidants (Basel). 2023 Feb 11;12(2):459. doi: 10.3390/antiox12020459 (PMC9952032; doi:10.3390/antiox12020459)
Supplement: Supplementary file 1 [file antioxidants-12-00459-s001.zip › antioxidants-2170460-supplementary.pdf]

Supplementary Table S1. Composition and nutrient levels of the basal diet (DM basis).

Items	Content
Ingredients / %	
Mixed silage	50.00
Corn	34.00
Soybean meal	5.50
Bran	8.00
Corn protein meal	1.00
Sodium bicarbonate	0.50
Premix [1]	0.50
Nacl	0.50
Total	100.00
Nutrient levels [2]	
ME /(MJ/kg)	14.73
CP /(g/kg)	151.14
RDP /(g/kg)	125.66
UDP /(g/kg)	85.95
Ca /(g/kg)	6.39
P /(g/kg)	5.49

ME, metabolic energy; CP, crude protein; RDP, rumen degrading protein; UDP, rumen undegraded protein. [1] Contained the following per kg of premix: VA 70 000~130 000 IU, VD 315 000~30 000 IU, VE ≥ 130 IU, Fe 0.4~0.8 g, Mn 0.5~1.0 g, Zn 1.5~3.0 g, Cu 0.1~0.2 g, Se 4~8 mg, Ca 80~160 g, P ≥ 10 g, NaCl 50~100 g. [2] ME was a calculated value, while the others were measured values.

Supplementary Table S2. Comparison of antioxidant capacity in different organs and tissues of Hu sheep [1] .

Items/organ	Kidney	Liver	Spleen	Pancreas	Intestinal Tract	Muscle	*p* Value
SOD, U/mL	834.55 [b]	2034.02 [a]	115.45 [e]	436.63 [c]	374.65 [cd]	237.35 [de]	<0.001
MDA, mmol/mL	1.83 [b]	1.66 [b]	2.53 [ab]	3.77 [a]	1.87 [b]	1.66 [b]	<0.001
GSH-Px, U/mL	626.58 [b]	113.38 [d]	922.82 [a]	215.78 [cd]	31.60 [c]	236.02 [cd]	<0.001
CAT, U/mL	27.47 [b]	113.85 [a]	2.57 [d]	4.10 [d]	10.10 [c]	3.22 [d]	<0.001
T-AOC, U/mL	0.02 [b]	0.05 [b]	0.03 [b]	0.05 [b]	0.05 [b]	0.63 [a]	<0.001

SOD, superoxide dismutase; MDA, malondialdehyde; GSH-Px, glutathione peroxidase; CAT, catalase; T-AOC, total antioxidant capacity. [1] Mean values with their standard errors of the mean (SEM), $n = 10$ in each group. [a,b,c,d,e] Mean values in the columns without a common letter differ ($p < 0.05$).

Supplementary Table S3. Effects of different temperament on amino acid contents of two kinds of muscles in Hu sheep (%).

Item	Clam Sheep	Nervous Sheep	SEM	*p* Value
RAA	1.9492*	1.7813	0.0424	0.029
NRAA	1.1493	0.9970	0.1179	0.414

RAA, reduced amino acid; NRAA, non-reducing amino acid. The values are expressed as the mean with standard error represented by vertical bars ($n = 5$). * indicated that there were significant ($p < 0.05$) differences between the two temperaments.

Supplementary Table S4. Effects of different temperament on contents of two kinds of muscle fatty acids in Hu sheep (%).

Item	Clam Sheep	Nervous Sheep	SEM	*p* Value
SFA	0.1668	0.3023**	0.0106	0.001
UFA	0.5615	0.8157	0.2127	0.318

SFA, saturated fatty acids; MUFA, monounsaturated fatty acids. The values are expressed as the mean with standard error represented by vertical bars ($n = 5$). ** indicated that there were significant ($p < 0.05$) differences between the two temperaments.
